# Gastric involvement: A rare extramedullary location of multiple myeloma

**DOI:** 10.1002/jha2.85

**Published:** 2020-08-24

**Authors:** Cláudia Pedrosa, Ana Rita Peixeiro, Patrícia Seabra, Luísa Regadas, Cláudia Casais, Cristina Gonçalves, Jorge Coutinho

**Affiliations:** ^1^ Department of Clinical Hematology Centro Hospitalar Universitário do Porto Porto Portugal

A 69‐year‐old caucasian man, ECOG‐1, was diagnosed with IgA/lambda and lambda free light chains multiple myeloma (MM), stage IIIB, ISS‐2/3, presenting with anemia, lytic bone lesions, hypercalcemia, and renal failure on dialysis.

He initiated treatment with bortezomib, dexamethasone (VD), epoetin, and zolendronic acid. Once creatinine recovered, thalidomide was added (VTD). He completed five cycles, obtaining partial response. Not being candidate for autologous transplant, he started on maintenance therapy with thalidomide and dexamethasone (TalDex).

Three months after VTD, progressive disease was observed with subcutaneous plasmacytomas. Cyclophosphamide (Cy) was added (CyTalDex) with regression of subcutaneous lesions.

At sixth CyTalDex, patient had 4‐day clinical course of melena with symptomatic anaemia. Upper digestive endoscopy (UDE) showed ulcerated gastric lesions with hemorrhagic foci and lesions with “volcano” morphology. Histopathology confirmed plasmablastic infiltration.

Patient started proton pump inhibitor and transfusion support, as he had no response to local hemostasis or systemic chemotherapy. Currently, patient awaits palliative gastric radiotherapy.

Gastric involvement presents in less than 1% of MM, associated with rapid progression and worse prognosis. Hemorrhagic presentation is rare. Therefore, in MM patient with upper gastrointestinal bleeding, gastric involvement must be considered. The promptly execution of UDE allowed the rapid diagnosis and to outline a therapeutic strategy.

**FIGURE 1:**
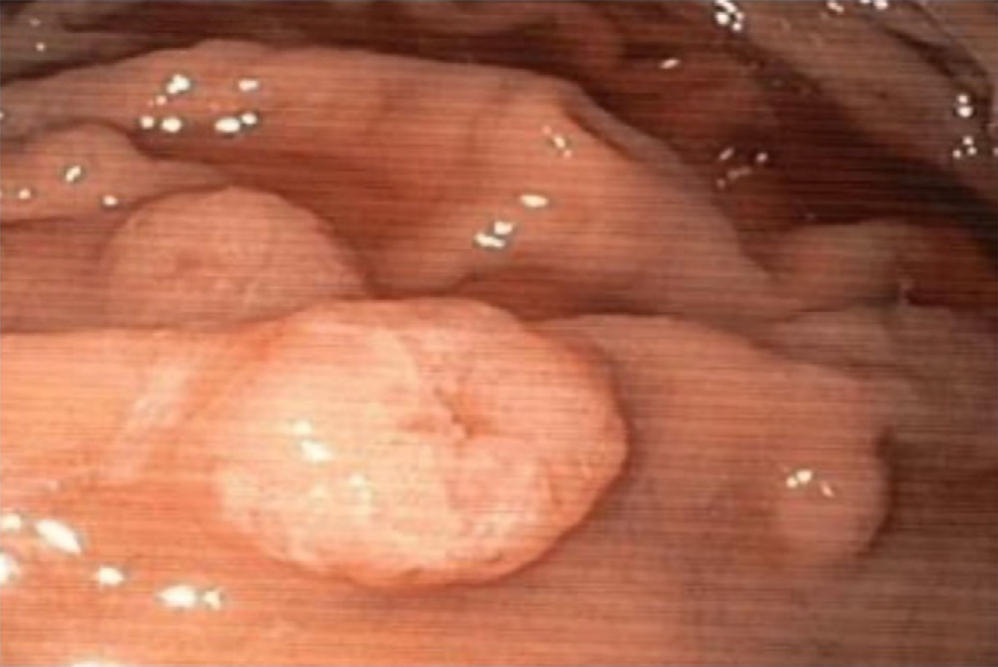
Gastric involvement with “volcano” morphology lesions.

**FIGURE 2:**
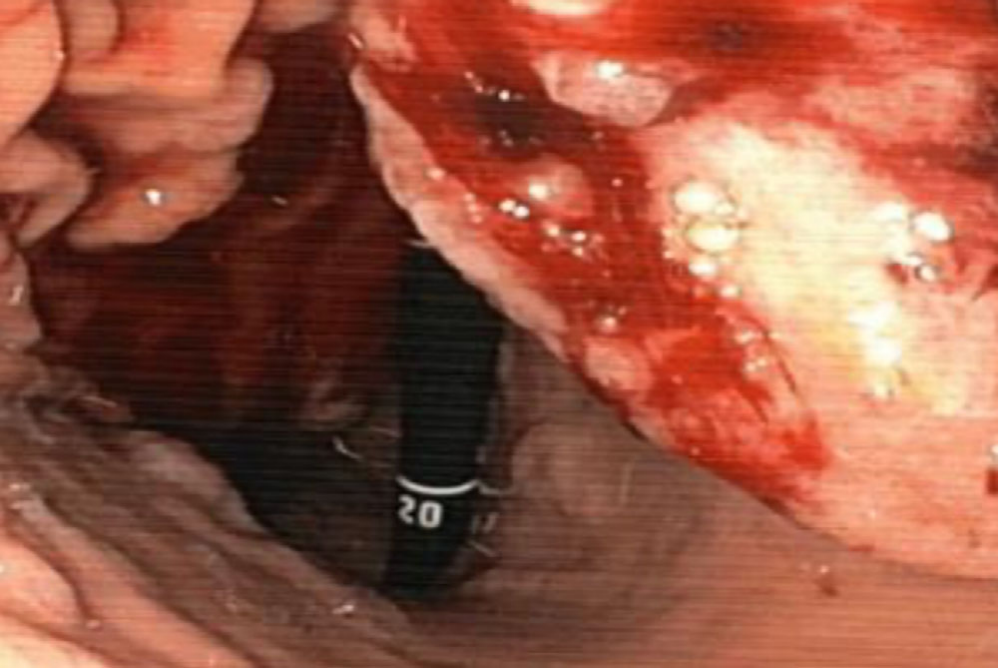
MM gastric involvement: ulcerated gastric lesions with hemorrhagic foci.

**FIGURE 3:**
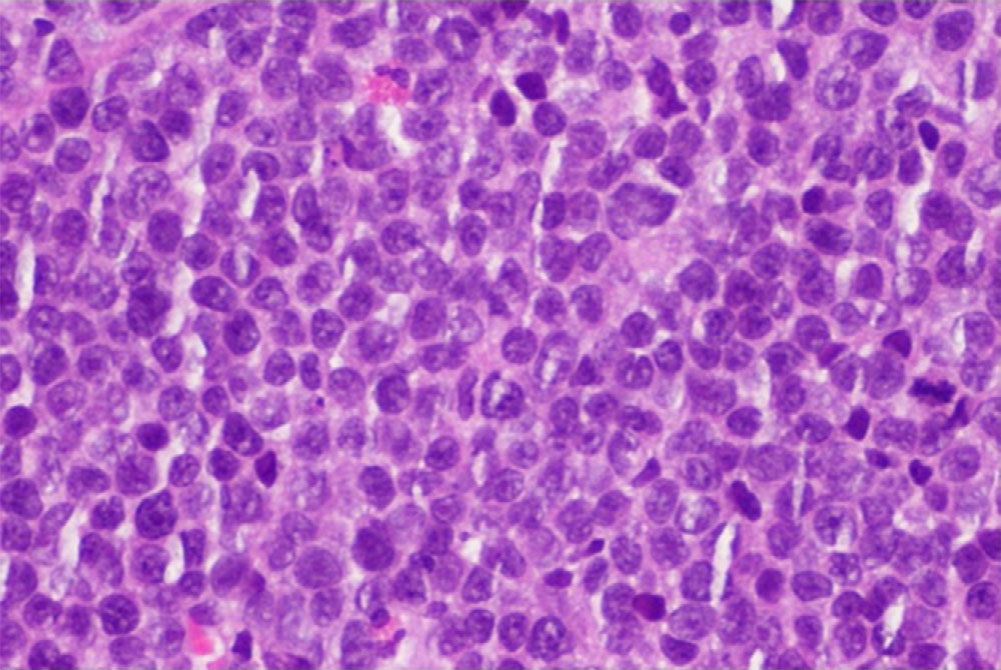
Gastric infiltration by plasma cells on histopathologic examination (plasmablastic morphology; hematoxylin‐eosin staining).

